# Multidimensional analysis of the learning curve for laparoscopic colorectal surgery in a regional hospital: the implementation of a standardized surgical procedure counterbalances the lack of experience

**DOI:** 10.1186/s12893-020-00975-6

**Published:** 2020-12-02

**Authors:** Ioannis G. Gkionis, Mathaios E. Flamourakis, Eleni S. Tsagkataki, Eleni I. Kaloeidi, Konstantinos G. Spiridakis, Georgios E. Kostakis, Athanasios K. Alegkakis, Manousos S. Christodoulakis

**Affiliations:** 1 Department of General Surgery, Venizeleio General Hospital, Leoforos Knossou 44, Heraklion, Crete Greece; 2 Medical School, University of Crete, Andrea Kalokerinou 13, Heraklion, Crete Greece

**Keywords:** Learning curve, Colorectal, Laparoscopy, Standardized surgical procedure, Without supervision

## Abstract

**Background:**

Although a larger proportion of colorectal surgeries have been performed laparoscopically in the last few years, a steep learning curve prevents us from considering laparoscopic colorectal surgery as the gold standard technique for treating disease entities in the colon and rectum. The purpose of this single centre study was to determine, using various parameters and following a well-structured and standardized surgical procedure, the adequate number of cases after which a single surgeon qualified in open surgery but with no previous experience in laparoscopic colorectal surgery and without supervision, can acquire proficiency in this technique.

**Methods:**

From 2012 to 2019, 112 patients with pathology in the rectum and colon underwent laparoscopic colorectal resection by a team led by the same surgeon. The patients were divided into two groups (group A:50 – group B:62) and their case records and histopathology reports were examined for predefined parameters, statistically analysed and compared between groups.

**Results:**

There was no significant difference between groups in the distribution of conversions (p = 0.635) and complications (p = 0.637). Patients in both groups underwent surgery for the same median number of lymph nodes (p = 0.145) and stayed the same number of days in the hospital (p = 0.109). A statistically important difference was found in operation duration both for the total (p = 0.006) and for each different type of colectomy (sigmoidectomy: p = 0.026, right colectomy: p = 0.013, extralevator abdominoperineal resection: p = 0.050, low anterior resection: p = 0.083).

**Conclusions:**

Taking into consideration all the parameters, it is our belief that a surgeon acquires proficiency in laparoscopic colorectal surgery after performing at least 50 diverse cases with a well structured and standardized surgical procedure.

## Background

Although the feasibility and oncologic efficacy of laparoscopic surgery for the treatment of inflammatory, benign and malignant disease entities in the colon and rectum have been demonstrated in randomized controlled trials [1–4], many surgeons are sceptical and avoid performing colorectal surgeries laparoscopically [5]. Four large prospective, randomized controlled trials, from North America, Canada and Europe, have been completed and have demonstrated that laparoscopic treatment of colon cancer yields oncologic results similar to those of open surgery, with no increased morbidity or mortality, and offers patients all the advantages of laparoscopic surgery [6–9]. Despite the fact that a greater adoption of laparoscopic colorectal surgery has been observed in recent years [10], the implementation of this technique is still progressing slowly [11]. According to recent statistical data, in England equal numbers of patients with pathology in the colon and rectum undergo open and laparoscopic surgery [12]. In other countries, the percentage of colorectal surgeries performed laparoscopically is much lower [13, 14]. The main reason for this lower percentage is the steep and long-term learning curve of laparoscopic colorectal surgery [15, 16].

The term "learning curve", which was first introduced by Hermann Ebbinghaus in 1885 in the study of Psychology of Learning and Theodore Paul Wright in 1936 for the aircraft industry to express the graphic representation of the mean rate of learning for a procedure, has been imported into laparoscopic colorectal surgery and is under investigation by several studies [17–21]. A learning curve is completed when the predefined variables reach a steady state and the outcomes are comparable with those in the literature [22, 23].

Although multiple parameters and numerous criteria have been taken into consideration to determine the adequate number of cases to achieve proficiency, a consensus has not yet been reached among surgeons [24–26]. No reliable framework for case selection during training is available, and consequently, the learning curve for laparoscopic colorectal surgery has not been conclusively analysed [27].

The aim of this study was to determine the safety and clinical outcomes of laparoscopic resection for colorectal disease entities performed by a single surgeon with no previous experience in laparoscopic colorectal surgery and to analyse the learning curve of a well-structured and standardized surgical procedure followed by a standardized postoperative protocol [28] using various parameters.

## Methods

From October 2012 to January 2019, 112 patients with pathology in the rectum and colon underwent laparoscopic colorectal resection at a regional general hospital (Venizeleio General Hospital of Heraklion in Crete), performed by a team led by the same surgeon (M.C.), who qualified for open surgery (> 300 colectomies) but had no previous experience with advanced laparoscopic procedures. Furthermore, laparoscopic colorectal operations were performed without the attendance or supervision of an experienced laparoscopic colectomy surgeon. Before beginning to perform colorectal resections laparoscopically, the surgeon (M.C.) became familiar with the cognitive aspects of this new procedure by watching operative videos, attending seminars and assisting during laparoscopic colorectal surgeries at specialized hospitals in this field. He obtained the necessary advanced laparoscopic surgery skills by training in animal models and virtual reality simulators.

Patients with locally advanced disease (T4 or bulky tumours), previous operations with a midline incision and ΒΜΙ > 35 were excluded from the study. The study population was organized chronologically according to the date of surgery and divided into two groups. The first group contained the initial 50 interventions, and the second group contained the following 62 patients. The case records and histopathology reports were examined for predefined parameters such as patient demographic data, location of the tumour, type of surgical procedure, conversion to open surgery, surgical time, distal and circumferential margins, number of harvested lymph nodes and total hospital stay. Mortality, surgical complications and oncological outcomes were also examined. The data collected (Additional file 1) were then statistically analysed and compared between these two groups.

The surgical time was calculated from the time of the first port placement to the time of wound closure, and the data for all laparoscopic surgeries were collected from the formal surgery records. Operations that were converted to open surgery were excluded from the surgical time analysis. The distal and circumferential margins and the number of harvested lymph nodes were obtained from the histopathology reports. Circumferential resection margins (CRMs) were defined as positive if malignant cells were found by microscopy at a distance of less than 2 mm.

### Preoperative preparation

All patients included in the present study underwent a preoperative preparation involving the following: extended medical report, physical examination, rectal exam, tests for the blood level of cancer markers, chest X-ray, thoracic and abdominal computerized tomography and total colonoscopy with biopsy. Patients with a tumour in the rectum underwent rigid rectoscopy and nuclear magnetic resonance imaging (MRI) of the rectum. If preoperative chemoradiotherapy was conducted for the patients with a rectal tumour, a repetitive MRI was performed.

### Surgical notes

All surgical procedures were performed by a team led by the same surgeon (M.C.) without the supervision of any surgeon specialized in laparoscopic colorectal surgery. The rest of the team consisted of two trainees, (one responsible for the camera and the other as a surgical assistant), and a single scrub nurse. The role of each member of the team was precisely defined to make everything work correctly.

General anaesthesia with epidural analgesia was the preferred mode of anaesthesia. A well-structured and standardized surgical procedure was implemented following oncological criteria, according to the principle of complete mesocolic excision (CME) and total mesorectal excision (TME) with central vascular ligation (CVL) [29–31]. For patients with a tumour in the lower rectum that was unsuitable for low anterior resection, the chosen surgical technique was extralevator abdominoperineal resection (ELAPE) with the patient in a jackknife position [32, 33].

### Postoperative protocol

All the patients in this study underwent a "fast track" postoperative protocol. Specifically, mobilization of the patients took place the next morning after the day of surgery. None of the patients had a nasogastric tube, so they were encouraged to drink liquids from the first postoperative day. The urinary catheter was removed the morning after the operation, with the exception of patients who underwent a low rectal resection or extralevator abdominoperineal resection. In these cases, the catheter was maintained for at least four days.

### Statistical methods

The mean, median and standard deviation (SD) were used to describe the continuous variables, such as the number of operation hours and days of hospitalization, while frequencies and %frequencies were used for discrete data. Independent samples t-test and the corresponding nonparametric Mann–Whitney test were used for two group comparisons. For comparisons of more than two groups, one-way ANOVA followed by Tukey's honest significant difference (HSD) post hoc test was used. Pearson's chi-square test was assessed to examine possible associations between two discrete variables. Box plots and scatter plots were used for graphical representation of the data. IBM SPSS Statistics 24.0 was used for statistical analysis of the data and an a = 0.05 limit was set for accepting the null hypotheses.

#### Results

From October 2012 to January 2019, a total of 112 patients underwent laparoscopic colectomies at Venizeleio General Hospital of Heraklion in Crete (Greece). Patients were formally divided into two groups named A and B. Group A was characterised as the training group and group B was characterised as the post-training group. In Table 1, the demographics of the patients (age and sex) are shown. There was no significant difference in the patients’ sex between groups A and B (p = 0.546) or their age distribution (p = 0.634).

**Table 1:** Sex and age distribution of patients between the training (A) and post-training group (B)

	Group				p
	Α		Β		
	n	%	n	%	
Sex					
1 (n = 48)	23	47.9	25	52.1	0.546
2 (n = 64)	27	42.2	37	57.8	
Age (10-years)					
≤ 50 (n = 13)	5	38.5	8	61.5	0.634
51–60 (n = 20)	8	40.0	12	60.0	
61–70 (n = 30)	11	36.7	19	63.3	
71–80 (n = 36)	19	52.8	17	47.2	
81 + (n = 13)	7	53.8	6	46.2	

Of a total of 112 patients who were initially selected for training and post-training laparoscopic surgery, nine (8.0%) were excluded due to conversion from laparoscopic to open surgery (Table 2).

**Table 2:** Distribution of operation conversion between the training group (A) and post-training group (B)

	Group				Total		
	Α		Β				
	n	%	n	%	n	%	p
Conversion							
No	46	92.0	57	91.9	103	92.0	0.99
Yes	4	8.0	5	8.1	9	8.0	(0.635)

### Patient diagnoses and operation types

**Fig. 1:**
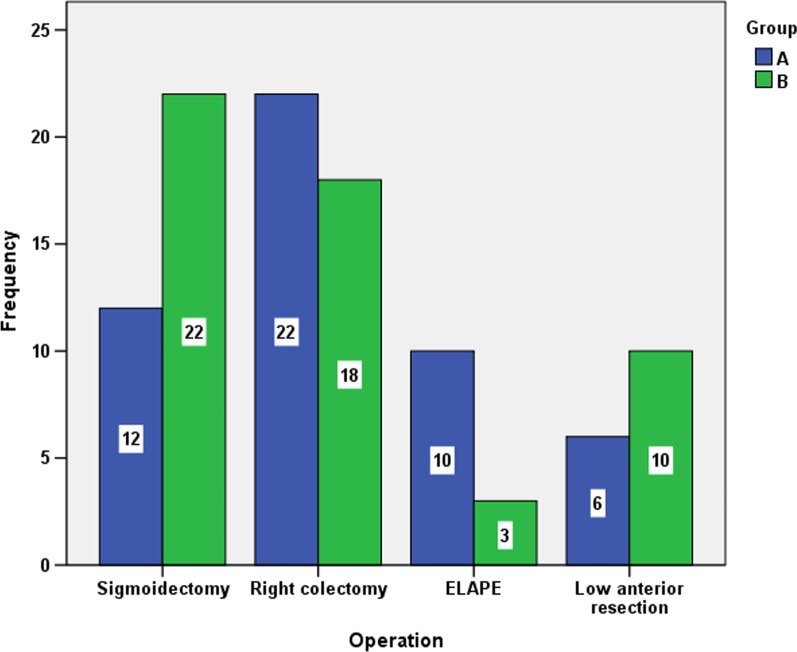
Distribution of operation types in groups A and B

The distribution of operation types in groups A and B is shown in Fig. 1. There was a higher number of sigmoidectomies in group B (n = 22) than in group A (n = 12), while the opposite pattern was shown for ELAPE operations, that is, n = 3 for group B vs n = 10 for group A (Fig. 1). A significant difference in the frequencies of operation types was found for group A and B patients (p = 0.045).

### Number of lymph nodes, days of hospitalization, duration of surgery and clinical characteristics between the pre- and post-training periods

In Table 3, the distribution of surgical margins and complications during the operation in the training (A) and post-training (B) groups are shown. Only 1 patient had positive margins, and this was recorded in group A. There was no significant difference in the type of margin proportions between the groups (p = 0.301). Operational complications were observed in 7 patients (6.8%). The distribution of complications was 4 cases in group A (8.0%) and 3 cases in group B (5.7%), which was not significantly different between groups (p = 0.637).

**Table 3:** Distribution of complications during the operation and type of surgical margins

	Group				
	Α		Β		
	n	n	n	**%**	**p**
Margins					
Free	49	98.0	53	100.0	0.301
Positive	1	2.0	0	0.0	
Complications					
No	46	92.0	50	94.3	0.637
Yes	4	8.0	3	5.7	

In Tables 4 and 5, the complications between training (A) and post-training (B) groups and for each type of surgery, regardless group, are depicted. For each of these 7 patients with complications, a laparotomy was performed after the initial laparoscopic colorectal operation. In addition, the patient in group B who exhibited anastomotic leakage after sigmoidectomy died during the postoperative period of the second surgery due to deterioration of the respiratory system. No other deaths were reported during the laparoscopic colorectal surgeries or the immediate postoperative period.

**Table 4:** Description of complications between the two groups

Group		
	Α	Β
	Complications	
Anastomotic leakage	2	1
Anastomotic stenosis	1	0
Ligature of the ureter	1	0
Lesion of the urinary bladder	0	1
Lesion of the iliac vein	0	1
Total number	4	3

**Table 5:** Description of complications for each type of surgery

Complications	Type of Surgery			
	Right colectomy	Sigmoidectomy	Low anterior resection	ELAPE
Anastomotic leakage	–	1	2	–
Anastomotic stenosis	–	1	–	–
Ligature of the ureter	1	-	–	–
Lesion of the urinary bladder	–	1	–	–
Lesion of the iliac vein	-	1	–	–
Total number	1	4	2	0

Patients in both groups were operated on for the same median number of lymph nodes. The median number with quartile range of the lymph nodes for group A was 20 (14–28), while for group B, it was 26 (16–34), showing no statistically significant differences (p = 0.145). Hospitalization days were not significantly different between the groups (p = 0.109), showing a median of 7 (5–8) for group A and 6 (5–7) for group B. The operation duration was shorter in the post-training group (group B) (median 4.0, 3.5–4.0 h) than in the training group (group A) (median 4.5, 3.5–5.5 h) (p = 0.006) (Table 6).

**Table 6:** Number of lymph nodes, days of hospitalization and operation duration between the training (group A) and post-training group (group B)

	Group										
	A					B					
	Mean	SD	Quartiles			Mean	SD	Quartiles			p*
			1st	2nd	3rd			1st	2nd	3rd	
Operation duration (hours)	4.5	1.2	3.5	4.5	5.5	4.0	1.4	3.5	4.0	4.0	0.006
Hospitalization (days)	7.9	4.7	5.0	7.0	8.0	6.2	1.9	5.0	6.0	7.0	0.109
Lymph nodes (number)	22.3	12.2	14.0	19.5	28.0	26.8	15.1	16.0	26.0	34.0	0.145

* Mann–Whitney test

### Surgical characteristics between the training group (A) and post-training group (B) per type of operation

When the type of operation was considered during the A vs B group comparison, although the median surgical duration was lower in group B than in group A, significance was present only for low anterior resection 4.5 (4.0–4.5) hours for group B vs 5.5 (4.5–5.5) hours for group A (p = 0.036). A tendency for significance was found for sigmoidectomy (p = 0.059). Group B showed a median of 3.5 (3.5–4.0) hours vs 4.5 (3.5–5.0) hours for group A (Table 7).

**Table 7:** Differences in surgical characteristics between the training group (A) and post-training group (B) per type of operation

		Group						
		A			B			
		Quartile			Quartile			
	Operation	1st	2nd	3rd	1st	2nd	3rd	P
Duration (hours)	Sigmoidectomy	3.5	4.5	5.0	3.5	3.5	4.0	0.059
	Right colectomy	3.5	4.0	5.0	3.5	3.5	4.0	0.286
	ELAPE	4.5	6.0	6.0	4.3	5.0	5.8	0.937
	Low anterior resection	4.5	5.5	5.5	4.0	4.5	4.5	0.036
Hospitalization (days)	Sigmoidectomy	6	6	8	5	5	7	0.557
	Right colectomy	5	7	8	5	6	7	0.697
	ELAPE	7	8	9	7	9	10	0.371
	Low anterior resection	6	7	8	6	6	7	0.713
Lymph nodes (number)	Sigmoidectomy	14	17	27	12	23	33	0.901
	Right colectomy	18	22	28	20	28	36	0.240
	ELAPE	7	14	16	16	18	37	0.077
	Low anterior resection	15	25	29	20	28	34	0.713

### Timeline of the operation duration

**Fig. 2:**
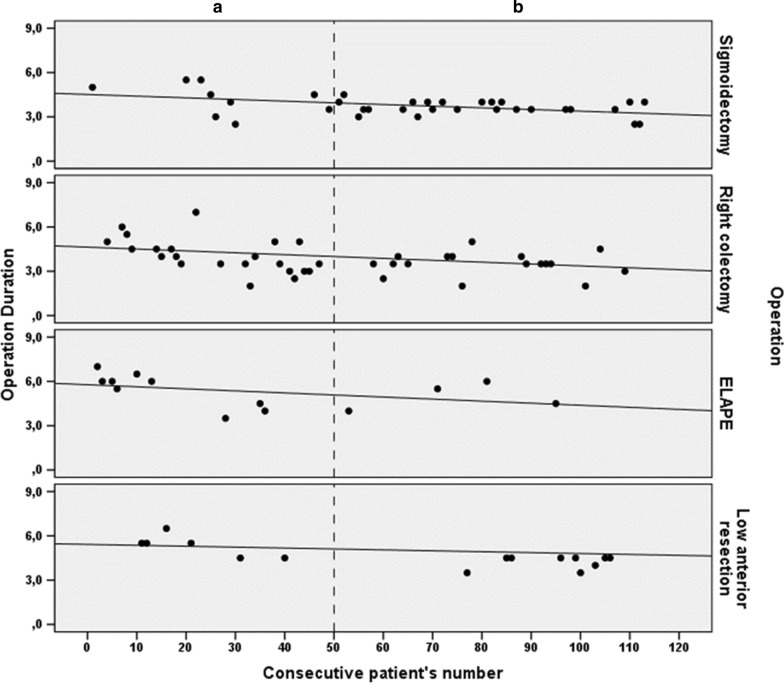
Timeline of the operation duration in hours vs the patients’ consecutive series for each operation type

An alternative approach is shown in Fig. 2. The timeline of the operation duration in hours is presented vs consecutive patients’ number for each of the operation types. Independent of the group (A or B), there was a significant decrease in operation time for sigmoidectomies (rs = -0.382, p = 0.026) as shown by the operation duration vs the patients’ consecutive series. The same pattern of decline was found for right colectomy operations (rs = -0.389, p = 0.013) and ELAPE (rs = -0.554, p = 0.050). A tendency towards decline was found for low anterior resection (rs = -0.446, p = 0.083).

## Discussion

Laparoscopic colorectal surgery is demanding because it requires an elevated level of technical skills. Although the feasibility and oncologic efficacy of laparoscopic colorectal surgery have been proven [1–4]**,** its implementation in daily surgical practice is still limited [11, 13, 14]. The main factor contributing to this limitation is the steep and long-term learning curve [15, 16]. In the literature, the number of cases needed to achieve proficiency in laparoscopic colorectal surgery varies enormously. Simons et al. reported a learning curve of 11–15 cases in a series of 144 patients in 1995 [34], whereas Tekkis et al. demonstrated a learning curve of 55 cases for right-sided colectomies versus 62 cases for left-sided resections [17]. In a multicentre analysis of 4852 cases, the learning curves varied from 87 to 152 procedures [27]. In other studies [15, 25, 26, 35, 36], the adequate number of laparoscopic resections ranged from 30 to 70 cases. As can easily be understood, there is no consensus among surgeons [24–26]. Therefore, the aim of this study was to evaluate the learning curve based on the initial outcomes of our first 112 operations using various parameters (Additional file 1).

The present study demonstrates that conversion rates do not differ significantly as the surgeon gains more experience. Our overall 8% conversion rate is in accordance with the 5–20% reported in the literature [37–39] and there was no significant change between the first 50 and the next 62 cases. Furthermore, no significant difference in the rate of surgical complications was identified between the two groups. Operational complications were observed in 7 cases among the total number of patients (6.8%), and the distribution was 4 cases in group A (8.0%) and 3 cases in group B (5.7%). Other reported series have concluded that at least 40–50 procedures are necessary to significantly lower the complication rate [20, 26, 34]. In addition, the 0.8% overall mortality rate and the 2.9% anastomotic leak rate for laparoscopic surgeries (3 cases of low anterior resection), which were detected in our study, are comparable with those in multicentre trials [37–39].

A very important parameter is the oncologic efficacy of the laparoscopic colorectal procedure. That goal, as represented by negative surgical distal and circumferential margins and an adequate number of harvested lymph nodes, can be reached early in the learning curve, as demonstrated in our research. The median and quartile range of lymph nodes for the training and post-training groups were comparable, showing no statistically significant difference. Furthermore, only one patient in the training group with a bulky tumour in the rectum locally expanded, despite neoadjunant chemotherapy and radiotherapy, had positive circumferential surgical margins. These results can be easily explained by taking into consideration the ample knowledge of anatomy and the respect for the rules of surgical oncology, which are credentials of the surgeon that are obtained by experience in open surgery. Inappropriate resection is not justifiable even in the training period, and oncological outcomes should not be compromised. For this reason, the completion of the operation laparoscopically does not constitute a purpose in itself.

The increasing number of cases performed laparoscopically did not alter the overall hospitalization of the patients between the two groups. The implementation of a well-structured and standardized surgical procedure followed by a standardized postoperative protocol ensures that all patients take advantage of the benefits of laparoscopic surgery [6–9]. COLOR II and Ivanov P et al. reported reduced hospitalization as a result of faster recovery in laparoscopically operated patients versus those who had an open surgery [40, 41].

The operation time in laparoscopic colorectal surgery is longer than that in open surgery even if the resections are performed by surgeons with experience in advanced laparoscopic procedures [42]. In our experience, the operating time decreases with the surgeon’s increasing experience, and it is a useful criterion for evaluating the learning curve. Specifically, there was a significant decrease in operation time for sigmoidectomies, right colectomy operations and ELAPE, and a tendency towards decline was found for low anterior resections. Our results are similar to those of Choi et al., who demonstrated a decline in the duration of laparoscopic sigmoidectomy after 30–42 cases [35].

## Conclusions

The key factor for accomplishing adequate oncologic resections laparoscopically and for shortening the learning curve is the implementation of a well-structured and standardized surgical technique followed by a standardized postoperative protocol. From our perspective, a surgeon acquires proficiency in laparoscopic colorectal surgery after performing at least 50 diverse cases. It is our belief that the results of this study will encourage and enable a larger number of surgeons to adopt laparoscopic surgery in daily clinical practice as the gold standard technique for treating diseases in the colon and rectum, even if they do not have previous experience or they work in a regional hospital.

## Supplementary information

**Additional file 1.** Data set created by the examination of the case records and histopathology reports of the 112 patients who underwent laparoscopic colorectal interventions.  

## Data Availability

The datasets generated and analysed during the current study are included in this manuscript and its supplementary information files.

## Abbreviations

M.C.: Manousos Christodoulakis
CRM: Circumferential resection margins
MRI: Magnetic resonance imaging
CME: Complete mesocolic excision
TME: Total mesorectal excision
CVL: Central vascular ligation
ELAPE: Extralevator abdominoperineal resection
SD: Standard deviation
HSD: Honest significant difference
